# Supplementary material for: Detection of Sulfur Dioxide by Broadband Cavity-Enhanced Absorption Spectroscopy (BBCEAS)
Source: Sensors (Basel). 2022 Mar 29;22(7):2626. doi: 10.3390/s22072626 (PMC9002574; doi:10.3390/s22072626)
Supplement: Supplementary file 1 [file sensors-22-02626-s001.zip › sensors-1631664-supplementary.pdf]

Detection of Sulfur Dioxide by Broadband Cavity Enhanced Absorption Spectroscopy (BBCEAS)

Supplementary Information

Ryan Thalman[1] , Nitish Bhardwaj[2], Callum Flowerday[2], and Jaron Hansen[2]
[1]Department of Chemistry, Snow College, Richfield, UT, USA
[2]Deparment of Chemistry and Biochemistry, Brigham Young, University, Provo, UT, USA
Correspondence: Ryan Thalman (ryan.thalman@snow.edu)

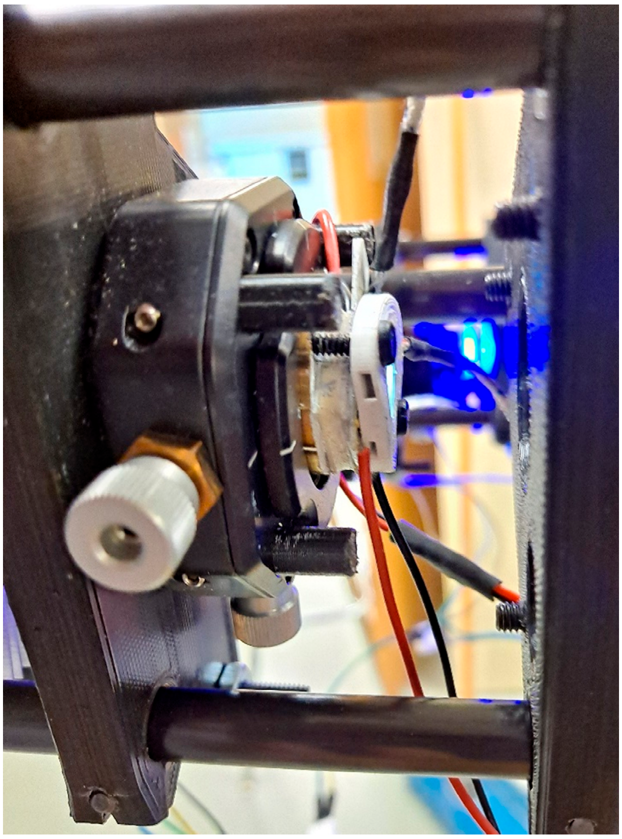

Figure S1: LED cooling mount in the cage system.

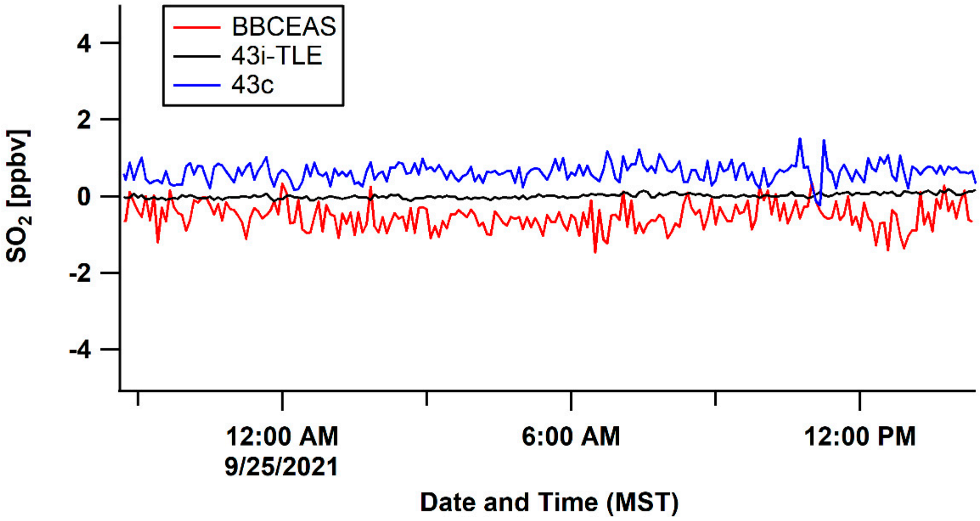

Figure S2: Ambient measurements of SO_2, averaged to 5-minute data for all three instruments.

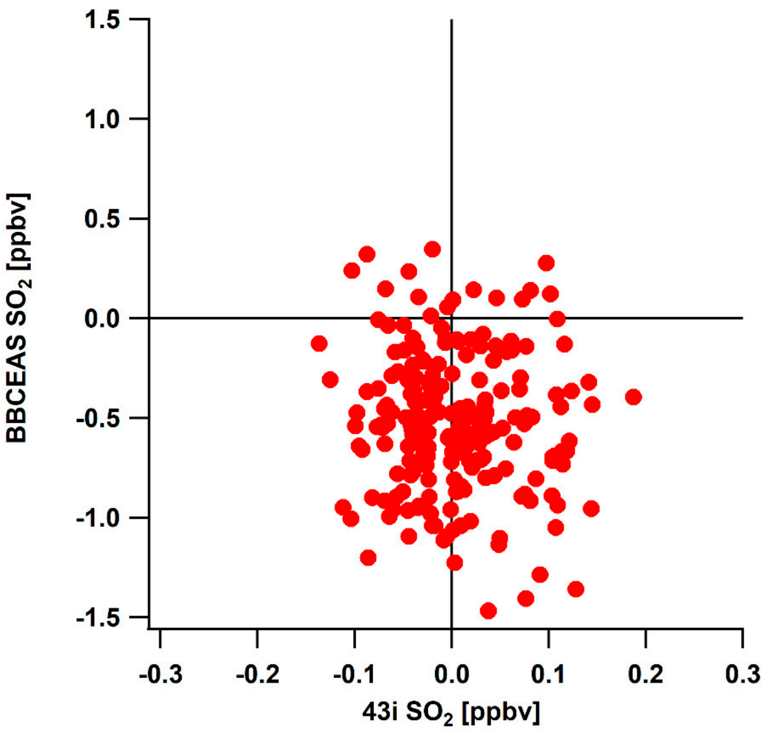

Figure S3: Correlation of ambient data from Figure S3 for BBCEAS measured [SO_2] relative to the 43i.

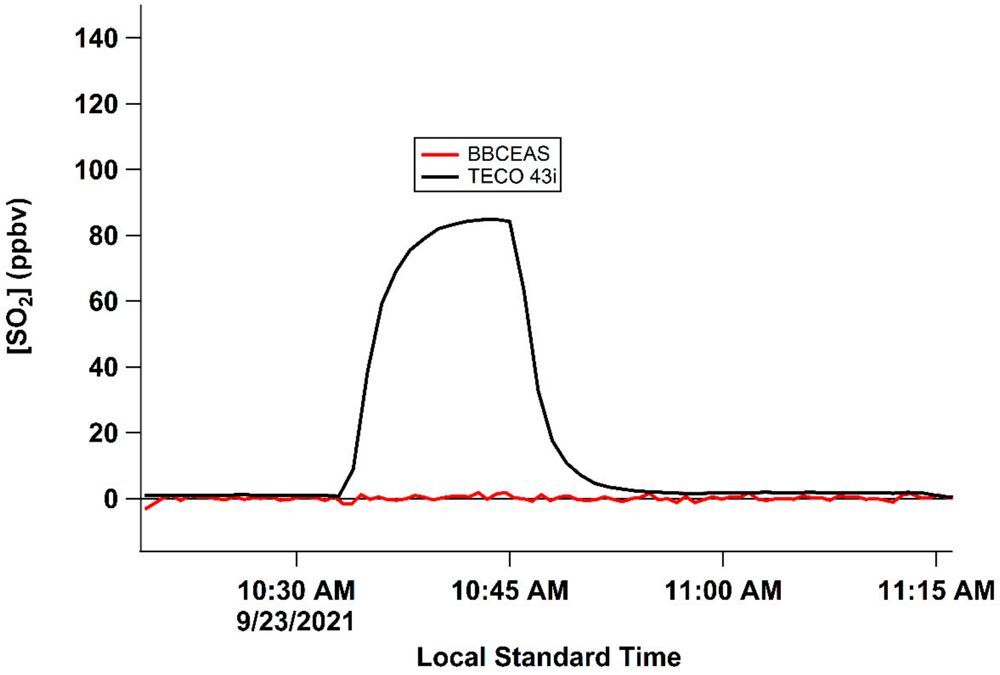

Figure S4: Response of BBCEAS vs. fluorescence detection in the presence of NO. 400 ppbv of NO is provided to the instruments with a measured SO_2 concentration of 85 ppbv in the Thermo 43i.
